# Schnitzler syndrome - a rare cause of chronic urticaria. Case report

**DOI:** 10.1093/omcr/omaf118

**Published:** 2025-07-27

**Authors:** Łukasz Moos, Aleksandra Kułakowska, Dorota Szydłowska, Weronika Chodak, Zenon Brzoza

**Affiliations:** Department of Internal Diseases, Allergology, Endocrinology and Gastroenterology, Institute of Medical Sciences, University of Opole, University Clinical Hospital, Al. Witosa 26, 45-401 Opole, Poland; Students’ scientific association “Alergos”, Institute of Medical Sciences, University of Opole, University Clinical Hospital, Al. Witosa 26, 45-401 Opole, Poland; Students’ scientific association “Alergos”, Institute of Medical Sciences, University of Opole, University Clinical Hospital, Al. Witosa 26, 45-401 Opole, Poland; Students’ scientific association “Alergos”, Institute of Medical Sciences, University of Opole, University Clinical Hospital, Al. Witosa 26, 45-401 Opole, Poland; Department of Internal Diseases, Allergology, Endocrinology and Gastroenterology, Institute of Medical Sciences, University of Opole, University Clinical Hospital, Al. Witosa 26, 45-401 Opole, Poland

**Keywords:** Schnitzler syndrome, chronic spontaneous urticaria, autoinflammatory disease, anakinra

## Abstract

Schnitzler syndrome (SchS) is a very rare acquired systemic disease that has many similarities to hereditary autoinflammatory syndromes. The condition is characterized by the presence of monoclonal gammopathy and chronic urticaria. In this case report, a 64-year-old male patient with SchS was initially misdiagnosed with chronic spontaneous urticaria (CSU). The patient was treated with anakinra, showing good tolerance and no need for steroid therapy for 14 months. SchS is often underdiagnosed and presents symptoms such as prolonged urticarial wheals and systemic manifestations. The differential diagnoses include mastocytosis, urticarial vasculitis, and autoimmune diseases. The diagnostic criteria were elevated CRP levels, neutrophilic skin infiltration, leukocytosis, and abnormal bone remodeling on scintigraphy. Treatment options include highly effective interleukin-1 blockade therapies such as anakinra, canakinumab, and rilonacept. This case emphasizes the importance of a thorough differential diagnosis of chronic urticaria and encourages clinicians to participate in the SchS database for improved recognition and management.

## Introduction

Schnitzler syndrome (SchS) is a recurrent urticarial rash with monoclonal gammopathy. Other possible symptoms include fever, musculoskeletal symptoms, lymphadenopathy, hepatosplenomegaly, and malaise. Laboratory tests can reveal signs of inflammation, such as leukocytosis, elevated CRP levels, acute-phase protein concentrations, and chronic disease anemia. The complement factor concentration is either within the normal range or increased. The exact pathogenesis of this disease remains unclear, but it involves excessive secretion of interleukin (IL)-1, IL-6, and IL-17 [1]. Almost 500 cases have been reported, mainly among Caucasians [2, 3]. The diagnosis is based on the Lipsker or Strasbourg diagnostic criteria (Table 1). According to Strasbourg, the obligatory criteria include chronic urticarial rash and monoclonal IgM or IgG (IgG isotypes remain rare at diagnosis). The minor criteria included recurrent fever, evidence of abnormal bone remodeling (which may not necessarily be painful), neutrophilic dermal infiltrate on skin biopsy, leukocytosis, and elevated CRP levels. The likelihood of diagnostic increases if monoclonal IgM is present. According to Lipsker, the major criteria remain unchanged; however, the minor criteria also consider arthralgia/arthritis, lymphadenopathy, hepatosplenomegaly, and elevated erythrocyte sedimentation rate [4].

**Table 1:** Strasbourg diagnostic criteria” for Schnitzler syndrome (CRP—C-reactive protein, Ig—Immunoglobulin) [4].

Obligate criteria	Minor criteria	Definite diagnosis	Probable diagnosis
Chronic urticarial rash	Recurrent fever	Both obligate criteria	Both obligate criteria
Monoclonal IgM or IgG	Evidence of abnormal bone remodeling with or without bone pain	At least two minor criteria for IgM positivity	At least one minor criterion for IgM
	A neutrophilic dermal infiltrate on a skin biopsy	At least three minor criteria for IgG positivity	At least two minor criteria for IgG positivity
	Leukocytosis		
	Elevated CRP		

Conventional therapies, including antihistamines, anti-inflammatory drugs, and immunosuppressive drugs, for systemic manifestations are typically ineffective or toxic. The IL-1 receptor antagonist anakinra is among the first-line therapies for treatment and has been found to rapidly control all the symptoms of this disease [2, 4]. In contrast to patients with chronic spontaneous urticaria (CSU), those with SchS are at increased risk of lymphoproliferative diseases, such as Waldenstrom’s macroglobulinemia and lymphoplasmacytic lymphoma [3].

## Case report

**Figure 1:**
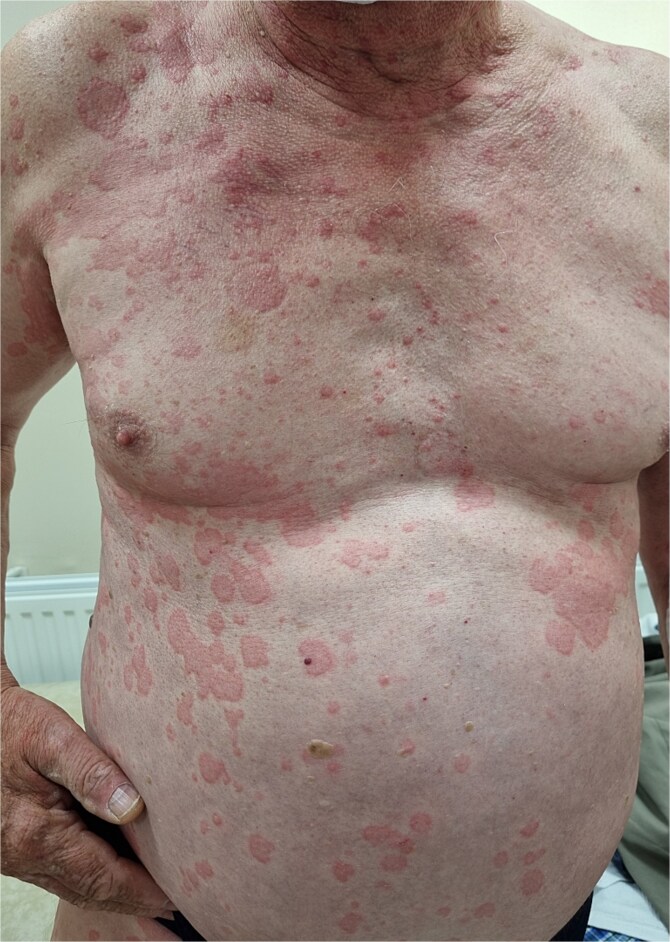
Patient’s skin at the admission.

**Figure 2:**
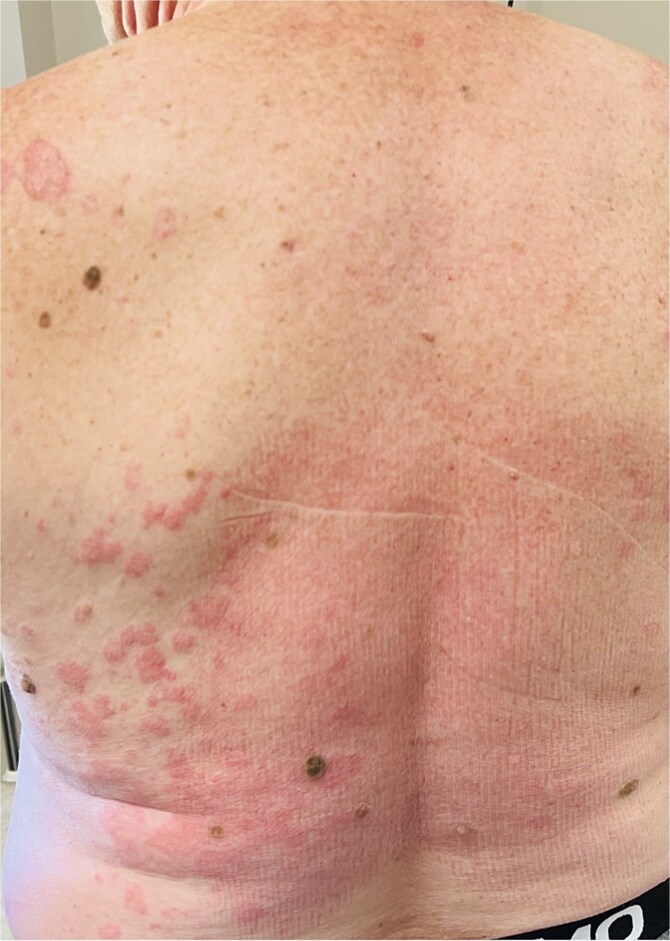
Patient’s skin at the admission.

**Figure 3:**
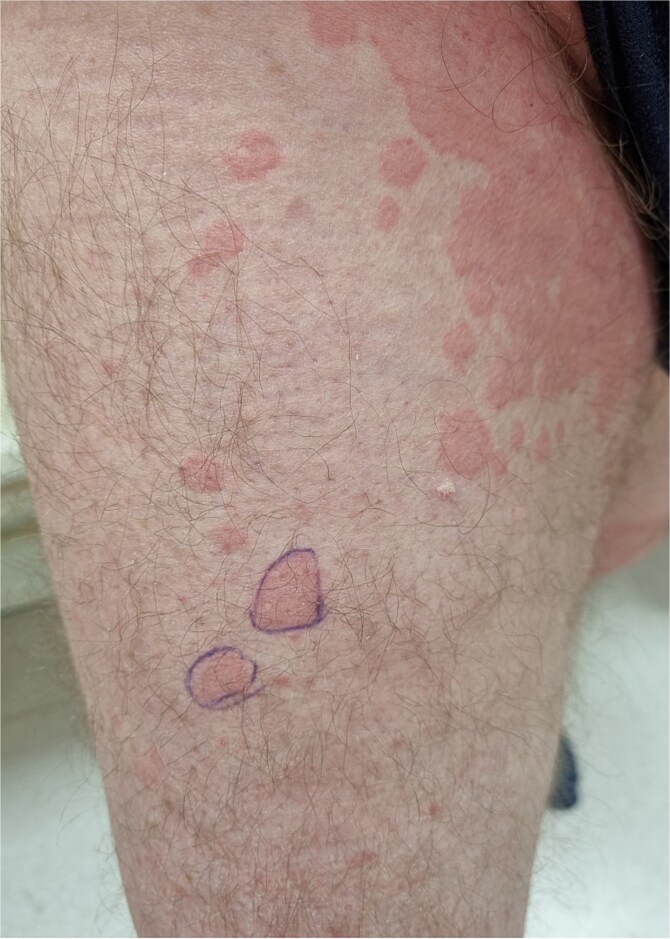
Presence of wheals 24 h after contouring on the patient’s skin.

**Figure 4:**
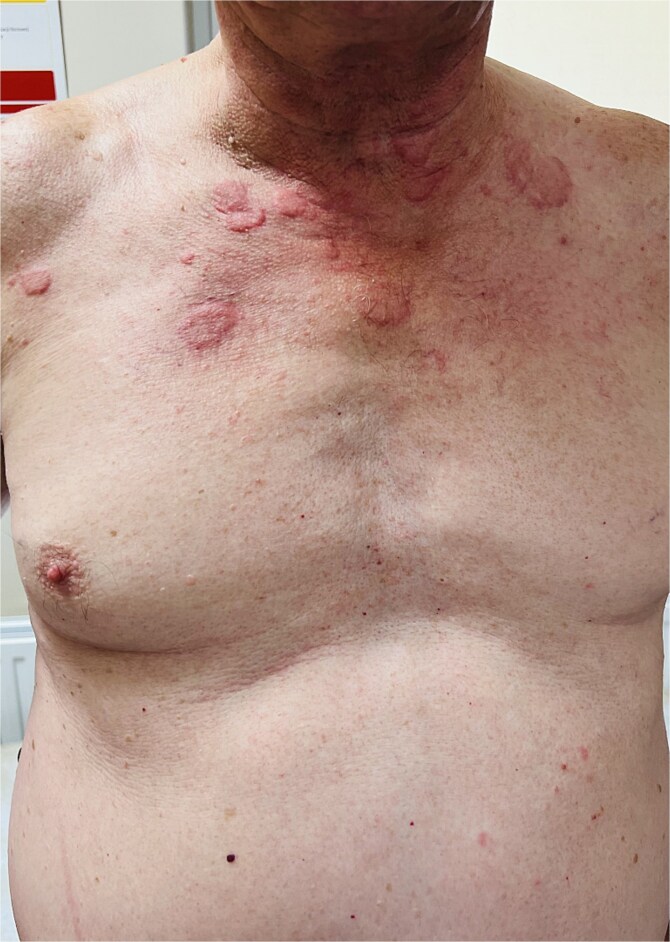
Improvements observed during systemic steroid therapy.

A 64-year-old man was admitted to the Department of Allergology because of an exacerbation of urticaria symptoms. In addition, the patient had hypertension, type 2 diabetes, glaucoma, and myelolipoma of the right adrenal gland. The patient reported arthralgia and frequent headache. He denied any allergies. The urticarial symptoms persisted for a year, with varying severity, but consistently recurring. The patient was treated for CSU with antihistamines at a quadruple dose per day (montelukast 10 mg once daily). Upon admission, the patient’s entire body was covered with merging, itchy wheals of various sizes (Figs 1 and 2) that persisted for about 24–48 h (Fig. 3) without angioedema. Systemic steroid therapy was initiated at a dosage of 10 mg in the morning and 10 mg in the evening, and the dose was carefully reduced by 5 mg every 7 days. Each time the steroid therapy was reduced to 10 mg of prednisone per day, symptom recurrence was observed (Fig. 4).

Laboratory tests revealed leukocytosis (13.76 G/l) with neutrophilia (9.31 G/l), elevated levels of CRP (15 mg/ml), IL-6 (31.4 pg/ml), IgM (3.08 g/l), glycated hemoglobin (7.9%), and total IgE (507.3 IU/ml). Low levels of IgA (0.57 g/l) and IgG (6.62 g/l) were noted, along with monoclonal gammopathy in the IgM class. Antibodies against the hepatitis C virus were positive, but there were no clinical signs of active infection, and the test for HCV RNA was negative.

Histopathological examination of a skin biopsy stained with hematoxylin–eosin revealed a normal epidermis and mixed cellular inflammatory infiltrates (lymphocytes and a few neutrophils) in the dermis around the superficial and deep plexus vessels. Interstitially scattered neutrophilic infiltrates were observed throughout the dermis. The microscopic change described was neutrophilic urticarial dermatosis.

The patient was referred to an immunology clinic where abnormal bone remodeling was diagnosed via scintigraphy, and SchS was confirmed. The patient was prescribed a daily subcutaneous injection of 100 mg of anakinra, which was well tolerated. To date, since the therapy began (14 months ago), the patient has not required steroid therapy. He was followed up at the hematology clinic; a bone marrow biopsy did not reveal any neoplastic infiltrates.

## Discussion

SchS is a rare but most likely highly underdiagnosed disease. According to Simon et al., the male/female ratio is 1.76, and the mean age at which the first symptoms appear is 51.6 ± 10 years [4]. The patient described in this report was a 64-year-old male with recurrent urticarial rash who had been treated for more than a year as CSU with poor response, although he was consulted by internal medicine doctors, dermatologists, and an allergologist.

The pathogenesis of SchS remains largely unknown. The similarity of the clinical presentation to cryopyrin-associated periodic syndromes (CAPS) in children has drawn scientists’ attention to the possible involvement of the nucleotide-binding oligomerization domain (NOD)-like receptor family pyrin domain containing 3 (NLRP3). Patients with CAPS have a point mutation in the NOD region, which is the center of the NLRP3 molecule. This mutation results in the self-polymerization and over-production of IL-1b by the NLRP3 inflammasome [5, 6]. The late onset of SchS is suspected to be the result of the mosaicism of NLRP3 in the myeloid lineage; there are 2 cases found by de Koning et al. [7]. However, the study by Lourvier et al. [8] was based on 40 cases of SchS showed the absence of NLRP3 somatic mutations or VEXAS-related UBA1 mutations. It is possible that patients with NLRP3 mutations present with the onset of NLRP3-autoinflammatory disease.

The presence of urticarial wheals is a common symptom in both SchS and CSU. In our case, the duration of the urticarial wheal was longer (24–48 h) than that of the CSU [9]. Additionally, there was a manifestation of joint pain, frequent headaches, and febrile conditions, which are commonly associated symptoms of this syndrome. Furthermore, no angioedema was observed [2]. Urticaria-like skin lesions may also be present in mastocytosis. A positive Darier sign, characteristic pigmented lesions, and elevated serum tryptase levels are the distinguishing features of mastocytosis, which is not observed in SchS. In addition, the presence of monoclonal gammopathy does not support the diagnosis of mastocytosis. A skin biopsy typically reveals an abnormal aggregation of mast cells in mastocytosis. [7].

SchS can be differentiated from familial Mediterranean fever, familial autoinflammatory cold reaction syndrome, and neonatal multisystem inflammatory disease (NOMID); however, the symptoms of these syndromes often appear at a younger age in children and adolescents [10].

Urticarial vasculitis, especially the hypocomplementemic variant (hypocomplementic urticarial vasculitis, HUV), can resemble SchS due to rash, fever, and joint pain. Cutaneous biopsy shows features of leukocytoclastic vasculitis manifested by signs of vessel damage involving postcapillary venules vasculitis, with fibrinoid necrosis of the small vessel walls, which is usually not the case in SchS. Complement consumption and anti-Clq antibodies are present, which is not observed in SchS [4]. HUV is distinguished by low complement levels and often presents with systemic involvement affecting organs, such as the kidneys, lungs, and gastrointestinal tract, thereby making it more severe. Normocomplementemic urticarial vasculitis, on the contrary, occurs with normal complement levels, but in contrast to SchS, it is typically limited to the skin, without significant systemic manifestations [11]. In our patient, the clinical picture and additional tests indicated SchS, which was diagnosed according to the Strasbourg diagnostic criteria. The patient met two mandatory and two minor criteria (elevated CRP level, neutrophilic infiltration in the skin confirmed by biopsy, and leukocytosis) [4]. Neutrophilic urticarial dermatosis described in skin biopsy is a rare form of dermatosis, and its most common associated diseases, apart from SchS, include Still’s disease, lupus erythematosus, and intermittent cryopyrin-related syndromes. However, during the diagnostic process, chronic infections, autoimmune diseases, and malignancies should be ruled out.

Patients with SchS are usually corticosteroid-sensitive but corticosteroid-dependent, requiring high dosages with moderate or specific effects, and drug reduction often leads to symptom recurrence. Long-term use of high-dose systemic steroid therapy is detrimental due to complications [3, 4, 12]. The patient described in this case report had poor initial diabetes control based on their glycated hemoglobin levels. In the described case, each reduction in the prednisone dosage to 10 mg per day resulted in symptom recurrence. In the case described by Bossard et al. [12], symptoms were controlled at a dose of 1 mg/kg body weight and recurred when attempts were made to reduce the dose to 20 mg/day [12]. De Koning et al. reviewed 281 cases and reported the effectiveness of 35 therapies. IL-1 blockade was the most effective option [3]. Among the possible drugs, anakinra, rilonacept, and canakinumab are also available [4, 13–15]. Anakinra, a recombinant human IL-1 receptor antagonist, is a first-line drug for treating SchS. It has been shown that 95% of patients achieve complete remission of their symptoms [13]. The mean treatment duration was 60 months [16]. The drug acts on the inflammasome and inhibits IL-1 activity. A reduction in IL-6 expression was also observed [5]. Another highly effective treatment is canakinumab (human anti-IL-1β antibody), with more than 90% effectiveness and a comparable safety profile [3, 12, 14]. Rilonacept is a dimeric fusion protein that blocks IL-1α, IL-1β, and IL-1 receptors. In a prospective study, rilonacept resulted in a rapid response and a sustained and significant improvement in health with good tolerability in 8 patients [15].

Matsuda et al. [17] after analyzing a population of individuals diagnosed with SchS [18] proposed a three-step therapeutic algorithm. The first step involves colchicine, and the second step involves nonsteroidal anti-inflammatory drugs (NSAIDs) and hydroxychloroquine. Targeted therapies against IL-1 and IL-6 are considered in the third step. They noted a higher efficacy of colchicine in the Japanese population than the 20% effectiveness mentioned by de Koning [3].

Each case of chronic urticaria, which is particularly poorly responsive to standard treatment, requires a thorough differential diagnosis. This encouraged all clinicians to be included in the SchS database.
